# Machine learning driven optimization of compressive strength of 3D printed bio polymer composite material

**DOI:** 10.1371/journal.pone.0330625

**Published:** 2025-08-28

**Authors:** R. S. Jayaram, P. Saravanamuthukumar, Ahmad Baharuddin Abdullah, Ramalingam Krishnamoorthy, Sandip Kunar, Xu Yong, S. Prabhakar

**Affiliations:** 1 Department of Mechanical Engineering, Amrita Vishwa Vidyapeetham, Nagercoil, Tamil Nadu, India; 2 School of Mechanical Engineering, Engineering campus, Universiti Sains Malaysia, Nibong Tebal, Penang, Malaysia; 3 Department of Sustainable Engineering, Saveetha School of Engineering, Saveetha Institute of Medical and Technical Sciences, Chennai, Tamil Nadu, India; 4 Department of Mechanical Engineering, Aditya University, Surampalem, Andhra Pradesh, India; 5 School of Artificial Intelligence and Smart Manufacturing, Hechi University, Yizhou, China; 6 School of Mechanical Engineering, Wollo University, Dessie, Ethiopia; IIIT Kurnool: Indian Institute of Information Technology Design and Manufacturing Kurnool, INDIA

## Abstract

3D printing has brought significant changes to manufacturing sectors, making it possible to produce intricate, multi-layered designs with greater ease. This study focuses on optimizing the compressive strength (CS) of functionally graded multi-material (PLA/Almond Shell Reinforced PLA) which is fabricated with the aid of the FFF process, a widely used additive manufacturing technique. Six different machine learning models (ML) were utilized to estimate CS using key process parameters, namely print speed (PS), layer height (LH), and printing temperature (PT). Among six ML models, Polynomial Regression (PR) performed best, with an R^2^ of 0.88 and the lowest error metrics (MAE = 1.38, RMSE = 1.9, MSE = 3.6). SHAP analysis indicated that PS is the most influential parameter, followed by LH. PR predicted optimal parameters (PS = 19 mm/s, LH = 0.1 mm, PT = 216°C) and yielded a predicted CS of 36 MPa, which was experimentally validated as 34.8 MPa with a low error of 3.44%. Also, the PR outperformed the traditional Taguchi method, which predicted a CS of 33.74 MPa, showing a 7.5% improvement and lower error. This demonstrates that PR-based ML optimization offers better accuracy and improved mechanical performance, making these FGMs suitable for various consumer applications.

## 1. Introduction

3D printing, or Additive Manufacturing (AM), refers to a contemporary method of producing parts by building them in successive layers guided by a digital model. AM methods are known to be adaptable and flexible in their designs and can be tailored for a myriad of industrial applications [1,2]. Among the various types of methods of AM, FFF is perhaps the most utilized due to its cost effectiveness. FFF constructs objects by extruding a thermoplastic material through a heated nozzle in a layer-by-layer fashion. This method provides greater design and improved mechanical performance as compared to traditional manufacturing methods [3,4]. Derived from lactic acid, PLA (Polylactic Acid) is a sustainable and degradable polymer widely employed in FFF techniques. It has mechanical properties similar to that of polyethylene terephthalate but with a much lower continuous use maximum mold temperature. Its biodegradability and renewability means its uses span a broad spectrum of products, including packaging, medical devices and textiles. Moreover, PLA can be recycled through hydrolysis or remelting into its monomer form [5–7]. Despite these advantages, PLA suffers from brittleness and limited mechanical strength when subjected to load-bearing conditions, reducing its effectiveness in demanding engineering applications. To overcome these drawbacks, PLA is often reinforced with a variety of materials including ceramics, carbon fibers, natural fibers, and nanocomposites to enhance its mechanical strength and durability [8–10]. Almond (Prunus amygdalus L.) is widely grown, especially in Gulf regions, with large-scale production generating notable byproducts like almond husks. Despite containing about 31% cellulose, these husks are often discarded or burned as waste [11,12]. Notably, the incorporation of wood-derived reinforcements has shown promise due to their abundance, renewability, cost-effectiveness, and ability to improve PLA’s recyclability and environmental performance [13]. Recently, researchers have been focusing on developing multi-material composites using innovative wood-reinforced PLA. Among the stacking sequences studied, the 50:50 configuration exhibited superior mechanical and thermal properties [14].

Reinforcement could improve the attributes of PLA, but the enhancement techniques in FFF have more influence. The PS, LH, and PT all impact the strength and overall quality of the material. These factors can also provide increased defect reduction with improved bonding. In the best way the automated processing is a requirement for improved functionality due to the adjustments made to the discussed parameters [15–17]. Many researchers have used the Taguchi optimization technique due to its versatility and ease in a vast number of studies focused on determining optimal process parameters [18,19]. Artificial Intelligence opened doors in many domains and it is no different to ML models in process optimization. Recently, there is an observed tendency among researchers to combine ML frameworks with Taguchi for process parametric prediction and optimization, provoking more refined searches and advanced selection criteria. Often, these are subjected to comparison for analyzing the effectiveness and precision achieved using these alternative methods [20,21]. Jatti V S et al. (2021) optimally set the parameters of FFF with a nonlinear ML model using the desirability method. The ML model yielded better results than the desirability approach since it had the lowest output prediction error when validated against experimental results. In another study, a researcher combined the Taguchi Design of Experiments with an L25 orthogonal array, two-layer 15 neuron in neural network (NN), and optimized the parameters in terms of compressive and tensile strength to streamline the process. The infill density was the strongest contributor for both compressive and tensile strength. With little prediction error, the Artificial Neural Network (ANN) model proved its reliability; further improving the results with additional test data would only enhance this reliability [22]. Deshwal S et al. (2020) [23] successfully optimized the FDM process parameters using hybrid optimization techniques like GA-ANN, which achieved a maximum strength of 47.0212 MPa. The GA-ANN method demonstrated the highest accuracy of 99.89% in predicting the optimal parameters. Ozkul M et al. (2025) [24] demonstrated the capability of ML algorithms to accurately predict the mechanical properties of ABS specimens fabricated through FDM. The results showed that the KSTAR algorithm best predicted hardness and surface roughness with MAE values of 0.006 and 0.009, respectively, and R^2^ values up to 0.99. The Multilayer Perceptron (MLP) algorithm was most accurate for predicting tensile and flexural strength, with R^2^ values over 0.99. Infill density was the most significant factor for hardness (55.56%), tensile strength (80.02%), and flexural strength (77.13%), while layer thickness had a 70.89% influence on surface roughness. These results emphasize the potential of ML algorithms in optimizing FDM 3D printing processes.

Teharia R et al. (2020) [25] conducted the study on Improving process variables for FFF manufacturing of PLA tensile specimens using a combination of Taguchi design and ANN approaches. The R^2^ value of 0.92 and a minimum prediction error of 5.33% were obtained, based on the comparison between the ANN model outputs and experimental findings. Raffic Noor Mohamed M et al. (2025) [26] also developed a structured experimental layout employing the Taguchi L18 array was set up to investigate six FDM process variables including infill pattern, density, layer thickness, orientation, temperature, and raster angle. The DTs and KNN models were employed to optimize the process parameters of FDM. The results indicated that the DTs algorithm outperformed the KNN algorithm across all four output responses, demonstrating better performance on the model evaluation metrics. The study by Saad M S et al. (2022) [27] applied a hybrid approach of ANN and SOS to effectively reduce surface roughness during FDM printing. By adjusting parameters like layer thickness, printing speed, print temperature, and outer shell speed, the model achieved a surface roughness of 2.011 µm. This result pointed to that the ML model was 12.36% better than the traditional Response Surface Method (RSM). Additionally, Borah J et al. (2025) [28] employed ML techniques, specifically Ridge and Bayesian linear regression, to precisely forecast the mechanical strength and surface quality of printed PEEK parts, with both models attaining a high R^2^ value of 0.92. Subsequently, a GA was employed to optimize the process variables. The GA optimization resulted in a maximum elastic modulus of 1264.37 MPa, a minimum surface roughness of 5.95 μm, and a maximum strength of 64.93 MPa. These results highlight the effectiveness of ML for process optimization in FDM additive manufacturing.

Furthermore, Gotkhindikar N N et al. (2024) [29] combined ML with Taguchi analysis to optimize FDM process variables to improve the mechanical strength. The study instigated a deep neural network that achieved an accuracy of 88.46% and an RMSE of 0.3396, based on 256 experiments. Taguchi analysis further confirmed the impact of key variables such as layer thickness, print line width, nozzle diameter and print speed on mechanical properties. These results highlight the potential of advanced ML methods in optimizing additive manufacturing processes. Soundararajan R et al. (2025) [30] employed five ML models such as Naïve Bayes, Multilayer Perceptron, J48 (C4.5), RF, and RT to predict the surface quality of printed PLA Plus components by using FDM. Among these, both Random Forest (RF) and J48 performed well. However, the J48 algorithm emerged as the best due to its highest accuracy, superior ROC curve performance, and faster processing time. Key printing parameters, notably PS and LH, were identified as the most influential on surface quality, with nozzle temperature playing a minor role. These findings allow manufacturers to predict surface finish reliably from input parameters using ML approach. Tandon S et al. (2024) [31] evaluated six ML models such as LR, RF, GBS, EGBR, VR, and an ANN to predict flexural properties of graphene-reinforced PLA. Flexural strength values ranged from 59.959 MPa to 129.511 MPa, with model performance evaluated using R^2^, MAE, and RMSE. LR emerged as the best performer, achieving an R^2^ of 98.9% and consistently lower error metrics compared to the other models. Based on the reviewed literature, ML models clearly outperform traditional methods in optimizing FFF process parameters for polymer composites. These models accurately predict key mechanical and surface properties.

In this study, six ML models LR, PR, SVR, KNN, DTs, and RF were employed to predict the CS of multi-material bio composites made from extruded PLA and almond shell reinforced PLA using FFF process parameters such as PH, LH, and PT. Data for training the models were obtained from our previous study [33]. Model performance was validated using metrics including R^2^, RMSE, MSE, MAE, and Cp, and the outperforming model was then used to predict the optimal FFF parameters for maximum compressive strength. The results were compared with Taguchi optimization outcomes from prior study, highlighting the benefits of integrating Taguchi methods with ML for accurate FFF process parameter optimization.

## 2. Study materials and methods

### 2.1. Input materials

To fabricate the extruded filament, INGEOTM 3D850 PLA pellets (white) were utilized, which have an approximate density of 1.24 g/cm^3^. These PLA pellets were procured from Natur Tec Limited, USA. In addition, discarded almond shells, a by-product of the dry fruit processing industry, were processed to obtain the particles of Almond Shell.

### 2.2. Synthesis of almond shell particles

**Fig 1:**
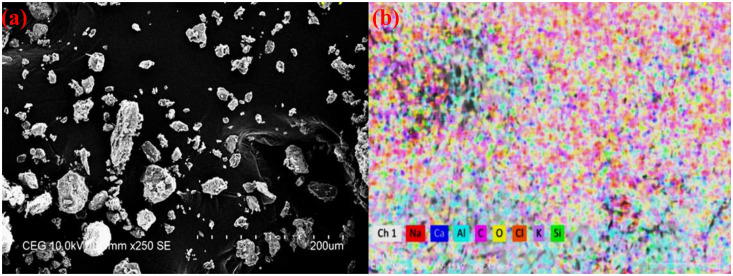
Morphological and compositional analysis of ASP particles: (a) SEM, (b) EDS mapping.

**Fig 2:**
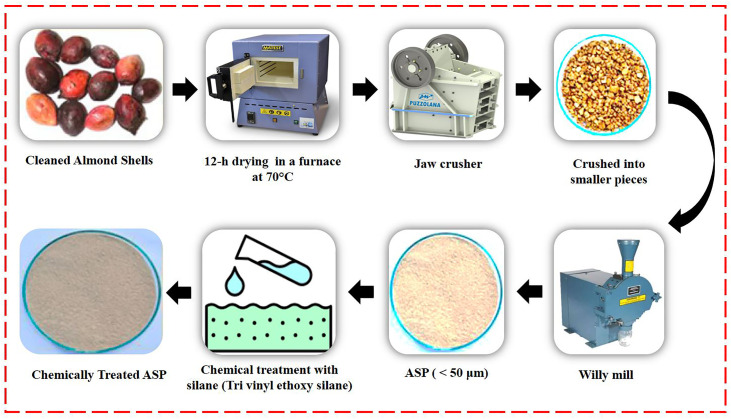
Preparation of ASP from wasted almond shells.

The discarded almond shells were initially washed to eliminate dirt, then dried at 70°C for 12 hours to remove any remaining moisture. They were then crushed using a jaw crusher and ground in a willy mill for uniform particle size. The particles were sieved to obtain sizes smaller than 50 μm. This particle size selection was made to ensure proper flow and processability in the FFF process. Specifically, the particle size needed to be smaller than both the minimum layer height (0.1 mm) and the nozzle diameter (0.127 mm) of the 3D printer used. Using finer particles prevents nozzle clogging and ensures uniform extrusion during printing. Morphological and size analysis of the ASPs was conducted, and the results are presented in Fig 1a. The almond shell particles (ASPs) predominantly exhibited spherical to irregular morphologies, with most particle sizes falling within the 30 µm to 50 µm range. This particle size range was also used in previous studies, which have shown that the improved dispersion within the virgin polymer matrix and enhance the mechanical effectiveness of composites [32]. Energy Dispersive Spectroscopy (EDS) analysis was conducted to determine the elemental composition of the ASPs which is displayed in Fig 1b. The result indicated that the C content of 55.21% and an O_2_ content of 40.31%. The calculated O_2_/C ratio is 0.73, which indicates a high cellulose content in the ASPs. This composition suggests that the almond shell particles have strong potential for load-bearing applications and can serve effectively as reinforcement in polymer composite materials. To improve bonding with the PLA matrix, the particles were chemically treated with 3% (w/w) silane (Tri vinyl ethoxy silane). These treated Almond Shell Particles (ASP) were used to produce reinforced PLA (AmdPLA) filament. The complete preparation process is displayed in Fig 2, and the constituent chemical makeup of ASP is listed in Table 1, highlighting the high cellulose content that enhances composite strength.

**Table 1:** Constituent chemical makeup of ASP [33].

Raw material	Cellulose (%)	Hemicellulose (%)	Lignin (%)	Extractives (%)	Other elements (%)
ASP	37.47	27.82	30.54	–	4.17

### 2.3. Extrusion of PLA and AmdPLA composite filaments

**Fig 3:**
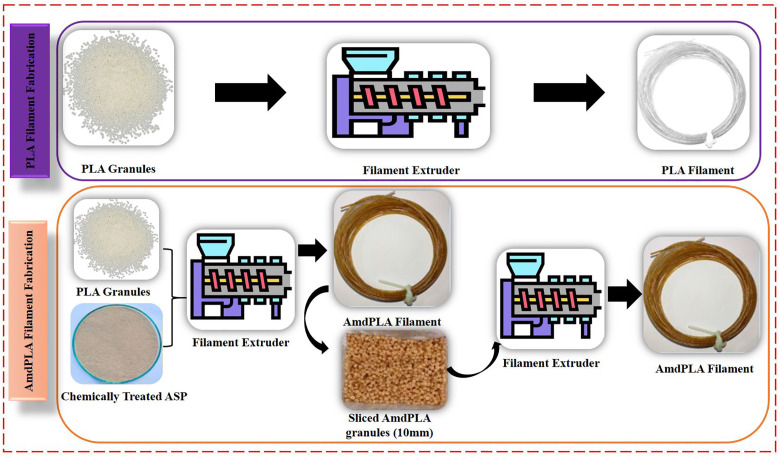
Illustration of fabrication process of PLA filament and AmdPLA filaments.

Using a single-screw bench-top filament extruder, filaments of pure PLA and AmdPLA were produced. The extrusion of pure PLA was conducted at a feed zone temperature of 135°C and an extrusion zone temperature of 150°C, with the screw operating at 220 rpm. To prepare the AmdPLA composite filament, 90% PLA granules were mixed with 10% Almond Shell Particles (ASP). A two-step extrusion method was adopted to ensure even distribution of ASP within the PLA. In the first step, the PLA and ASP were blended and extruded. The resulting filament was then chopped into 10 mm pieces and re-extruded in the second step to improve mixing quality. This repeated extrusion ensured that the ASP was well distributed in the virgin PLA base. The final extruded composite filaments had a uniform diameter of 1.75 mm ± 0.05 mm and were utilized as the feedstock for an FFF 3D printer (Model: Pratham 2.0, India). Fig 3 illustrates the complete fabrication process of PLA filament and AmdPLA filaments.

### 2.4. Preparation of functionally graded material combining PLA and AmdPLA

**Fig 4:**
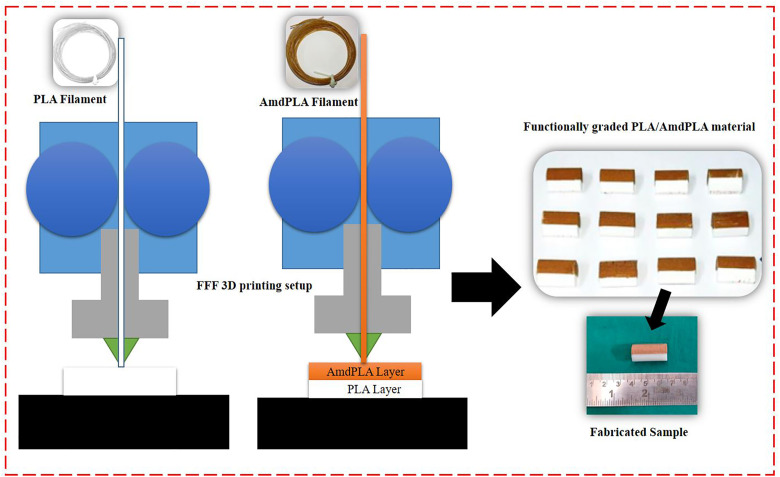
Illustration of the complete fabrication steps of multi-layer PLA/AmdPLA material.

Functionally graded components were fabricated using an FFF 3D printer with extruded filaments of virgin PLA and AmdPLA composite. Compression test specimens (12.7 mm diameter, 25.4 mm height) were printed horizontally as illustrated in Fig 3. The specimen design was built in CATIA and changed into an STL file. To create the functionally graded structure, the total number of layers was divided equally—half printed with neat PLA and the other half with AmdPLA. The filament was manually switched midway by pausing the printer. All printing parameters were kept constant throughout the process. The complete fabrication steps are illustrated in Fig 4.

### 2.5. Compression testing of prepared 3D printed sample

**Fig 5:**
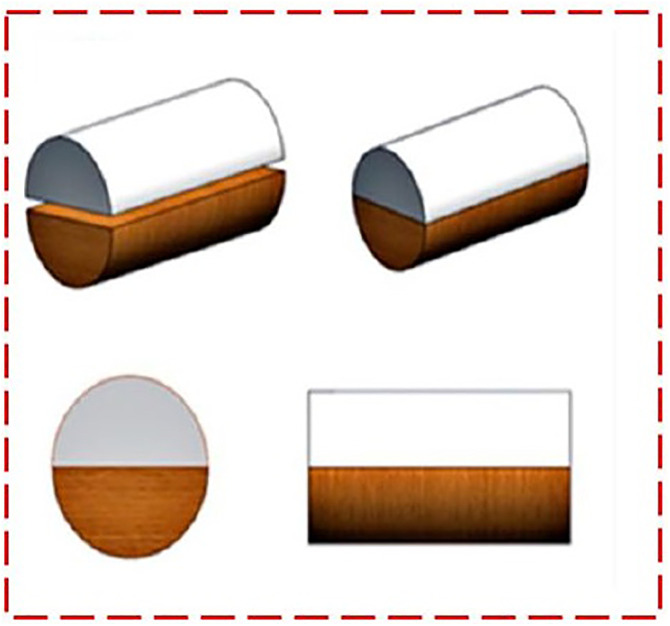
Illustration of 3D printed multi-layer PLA/AmdPLA sample for Compressive test.

The CS of the 3D fabricated PLA/AmdPLA samples was tested using a compression test. A digital Universal Testing Machine (UTM) equipped with compression capabilities was employed to apply load at a steady speed of 1 mm/min. The tests followed the ASTM D 695 standard for compression. The samples were designed using CATIA V5R20 software. Each specimen was placed in the machine in a vertical (axial) position, and the load was gradually applied to measure the peak compressive strength. Tests were done for different 3D printing parameters using the FFF method. The compression test sample is illustrated in Fig 5.

### 2.6. Machine learning

**Fig 6:**
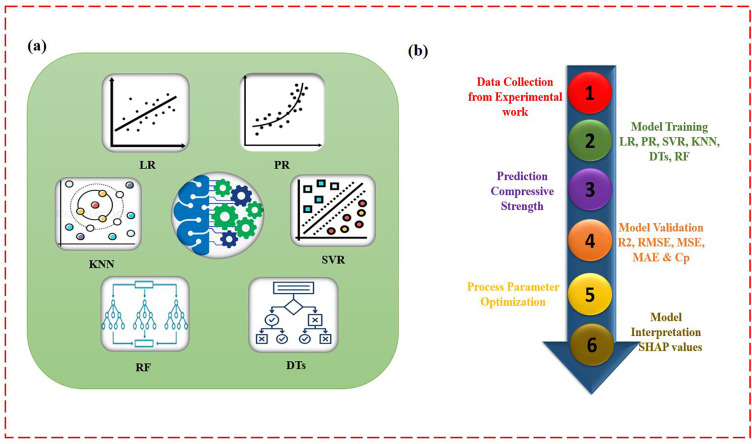
Illustration of (a) Six different ML techniques, (b) overall workflow of the predictive modelling process.

In this research work, experimental data set were composed from our previous studies. The input parameters and their resultant levels are listed in Table 2. ML models were developed and trained using Python in Google Colab (version 1.0.0, License: Apache 2.0), with the help of the Scikit-learn library. Six different ML models were applied to predict the CS, as illustrated in Fig 6a. Fig 6b shows the overall workflow of the predictive modelling process used in this study.

**Table 2:** FFF printing process parameters and corresponding level [33].

Sl. No	FFF Process Parameters	Unit	Level			
			1	2	3	4
1	Printing Speed (PS)	mm/s	10	20	30	40
2	Layer Height (LH)	mm	0.1	0.15	0.2	0.25
3	Printing Temperature (PT)	^o^C	190	200	210	220

### 2.7. Hyper parameters of ML models

Hyper parameters are crucial settings in ML models that must be defined before training, as they directly influence how the model learns from the data which is shown in Table 3. In PR, the key hyper parameter is the degree of the polynomial, which controls the model’s complexity too low leads to under fitting, while too high can cause overfitting. In SVR, the important hyper parameters include the kernel type (such as linear or RBF), C (regularization), and epsilon, which affect how the model handles complex relationships and margins. For KNN, the primary hyper parameter is the number of neighbour (K); small values may over fit, while large values may underfit, with typical values like 3 or 5 providing a balance. DT rely on hyper parameters such as without limit on depth, minimum samples required for splitting, and least requirement of leaf to control tree growth and avoid overfitting. In RF, key hyper parameters include estimators (the number of decision trees), automatic selection of features, and bootstrap, which impact accuracy and generalization by reducing variance through ensemble learning. Tuning these hyper parameters appropriately is essential to optimizing model performance across different types of datasets [34].

**Table 3:** Hyper parameter settings for ML Algorithms.

Sl. No	ML Models	Hyper Parameters
1	LR	Linear regression with intercept enabled and normalization turned off.
2	PR	PR of degree 2 without bias term.
3	SVR	SVR with RBF kernel, penalty C = 1.0, epsilon margin of 0.1, and automatic gamma scaling.
4	KNN	Using 5 nearest neighbours, uniform weighting, automatic algorithm selection, and leaf size of 30.
5	DTs	No limit on tree depth, splitting requires 2 samples, each leaf must have 1 sample, and no feature limit.
6	RF	Random Forest with 10 trees, unlimited depth, minimum 2 samples to split, leaf size 1, feature selection is automatic, with bootstrapping and fixed seed of 42.

### 2.8. Data collection

**Fig 7:**
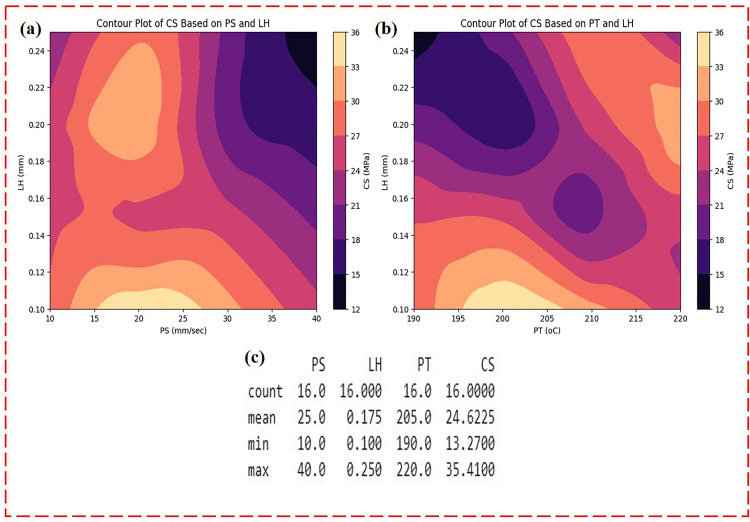
Dataset overview for ML modelling: (a) PS, LH vs CS, (b) PT, LH vs CS parameters, and statistical summaries (c), providing insights into the key trends in the dataset.

The dataset was utilized for both training and evaluating the ML models in this research work was obtained from our previous research [33]. In that study, experimental runs for the FFF process were planned using the L16 Taguchi orthogonal array, considering three factors (PS, LH and PT) at four levels. A total of 16 combinations were generated using Minitab software, and for each combination, compression test specimens were fabricated, with their compressive strength measured through compression tests. These 16 data points formed the basis for training and evaluating the ML models in this current study. To facilitate effective analysis, Fig 7 presents visualizations such as density contour plots (a, b), which show the distribution of the input and output. The collected data falls within specific parameter ranges: PS (10 mm/s to 40 mm/s), LH (0.1 mm to 0.25 mm), and PT (190°C to 220°C).

### 2.9. Model validation

Model validation involves assessing the ability of a trained model to accurately forecast the results based on input data. While training and testing are vital stages, validation ensures that the model can consistently produce accurate predictions. To validated the effectiveness of the model, various metrics are used, including R^2^, RMSE, MSE, MAE, and Cp. The R^2^ value varies between 0 and 1, where values approaching 1 signify excellent accuracy and strong predictive ability, whereas values close to 0 indicate weak prediction performance [35]. For RMSE, MSE, and MAE, lower values indicate better performance, as these metrics reflect the average prediction errors—smaller error values mean the predictions are in close agreement with the true outcomes [36]. Cp measures the linear correlation of actual versus predicted outcomes, with a Cp near 1 signalling a strong correlation and a good model. Cp is calculated separately for both the training and test data sets. When the Cp value for the test set exceeds that of the training set, it indicates that the model generalizes effectively and does not overfit. The noticeable decrease in Cp for test data prediction versus training data suggests overfitting, possibly due to noise in the data [37].


$${\text{R}}^{\text{2}}[\text{35}]=1-\frac{\sum {\left(y_{i}-\hat{y_{i}}\right)}^{2}}{\sum {\left(y_{i}-\overset{―}{y}\right)}^{2}}$$ (1)

where:

- $y_{i}$ = True values
- $\hat{y_{i}}$ = Anticipated values
- $\overset{―}{y}$ = Mean of true values

$$\text{RMSE}[\text{36}=\sqrt{\frac{1}{n}\sum {\left(y_{i}-\hat{y_{i}}\right)}^{2}}$$ (2)

$$\text{MSE}[\text{36}]=\frac{1}{n}\sum {\left(y_{i}-\hat{y_{i}}\right)}^{2}$$ (3)

$$\text{MAE}[\text{36}=\frac{1}{n}\sum \left|y_{i}-\hat{y_{i}}\right|$$ (4)

$${\text{C}}_{\text{p}}[\text{37}=\frac{\sum \left(y_{i}-\hat{y}\right)\left(\hat{y_{i}}-\overset{―}{\hat{y}}\right)}{\sqrt{\sum {\left(y_{i}-\hat{y}\right)}^{2}}\sqrt{\sum {\left(\hat{y_{i}}-\overset{―}{\hat{y}}\right)}^{2}}}$$ (5)

where:

- $\overset{―}{y}$ = Mean of actual values
- $\overset{―}{\hat{y}}$ = Mean of predicted values

## 3. Data analysis and discussion

### 3.1. ML model assessment on compressive strength (CS) prediction

**Fig 8:**
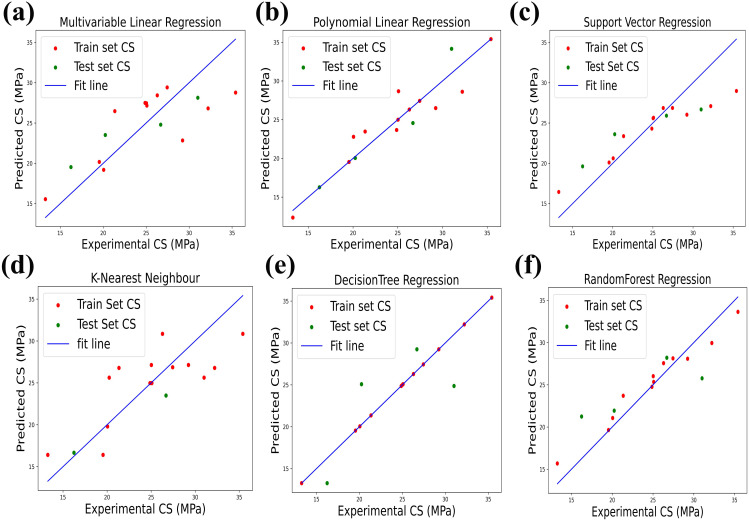
Experimental CS from dataset vs. Predicted CS by (a) LR, (b) PR, (c) SVR, (d) KNN, (e) DTs and (f) RF.

Fig 8 presents an assessment between the experimentally measured CS and the predicted CS values obtained from various regression models. The blue line represents the ideal fit (reference) line, serving as a benchmark to evaluate model performance models are considered more accurate when data points closely follow this line. The dataset is split into two groups: red points indicate the training set, and green points represent the test set. Both sets are used to assess the predictive ability and generalization of six ML models.

**Fig 9:**
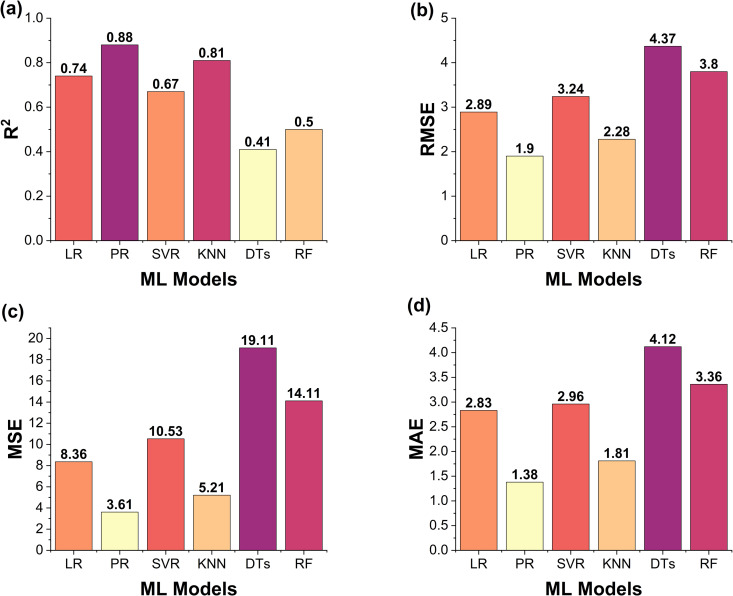
Quantitative evaluation of the six ML models on CS. **(a)** **R**^**2**^, **(b)** **RMSE,** **(c)** **MSE and** **(d)** **MAE.**

Among all models, PR shows the best performance, with most training and test points aligning closely with the reference line, indicating high predictive accuracy. KNN ranks next, as several test points also lie near the fit line. In contrast, LR, SVR, RF, and DTs show lower accuracy, in that order. Notably, DTs exhibit significant overfitting all training points lie perfectly on the fit line, but test points deviate widely, highlighting its poor generalization and the weakest performance among all models. Multiple performance indicators including R^2^, MAE, MSE, and RMSE were used to assess the performance of the ML models, as shown in Fig 9. Fig 9a presents the R^2^ values for all six models. Among them, PR achieved the highest R^2^ value of 0.88, indicating excellent prediction accuracy. KNN followed with a value of 0.81, showing good performance. LR and SVR recorded moderate R^2^ values of 0.74 and 0.67, respectively. In contrast, DTs and RF showed poor performance, with DTs having the lowest R^2^ value among all models.

Fig 9b–9d illustrate the error-based metrics (RMSE, MSE, and MAE). These metrics measure the difference between predicted and actual outcomes, where lower values show the better model effectiveness. The trend in these metrics aligns with the R^2^ scores: PR exhibited the lowest errors, while DTs had the highest, confirming its poor generalization ability. The above results show that PR is identified as the most effective model for predicting the CS of multilayer PLA/AmdPLA functionally graded materials fabricated through the FFF 3D printing process. Its superior performance further confirms the non-linear correlation between the FFF process input parameters and the CS output.

### 3.2. Pearson correlation of the ML models

**Fig 10:**
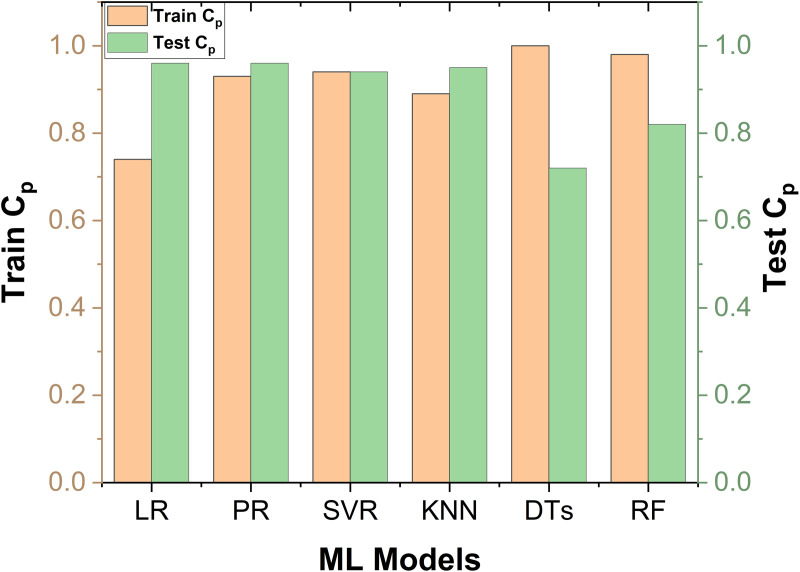
Comparison C_p_ for training and test sets across ML models.

Fig 10 presents a comparison of the train C_p_ and test C_p_ values for six ML models. The Cp statistic evaluates the connection between training and test data points, playing a vital role in determining the model’s reliability when predicting new, unseen data. Examining this relationship aids in assessing the model’s generalization capability. From the figure, it is evident that LR, PR, SVR, and KNN models did not exhibit overfitting. In these models, the test C_p_ value is consistently higher than the train C_p_ value, indicating that these models perform well on unseen data and generalize effectively based on the model performance.

In contrast, DTs and RF models show lower test C_p_ values compared to their train C_p_ values, suggesting overfitting. As seen in Fig 7e, 7f, DTs demonstrates clear overfitting while all training data points are perfectly aligned with the reference line, none of the test data points align, leading to a large difference between train C_p_ and test C_p_. On the other hand, the RF model shows some misalignment between the training data points and the reference line, though fewer points are perfectly aligned compared to DTs. As a result, the difference between train C_p_ and test C_p_ for RF is smaller than that of DTs, indicating less severe overfitting.

### 3.3. Model interpretation

**Fig 11:**
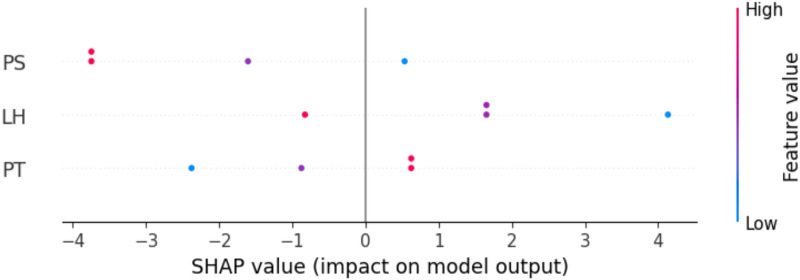
SHAP-based interpretation of influencing process parameters on compressive strength.

SHAP (SHapley Additive exPlanations) explains how much each the input parameters influence the output prediction based on the ML model. In a SHAP summary plot, each dot represents a prediction, and its colour shows the actual feature value (red = high, blue = low, purple = medium). Wider SHAP value spread for a feature means it has a stronger influence on the prediction, with features listed top to bottom in order of decreasing importance. Positive SHAP values show features that increase the predicted output, whereas negative SHAP values represent features that decrease it [38,39]. Fig 11 displays the SHAP summary plot, which visualizes how each feature PS, LH, and PT affects the model’s prediction of compressive strength across various instances. The PS feature exhibits the widest spread of SHAP values and located at top of the feature list, indicating that it is the most significant process parameter in predicting compressive strength. The PS feature has a mixed impact for some instances, decreasing PS values can either increase or decrease the output, while for other instances, high values tend to reduce the predicted CS, as reflected by their negative SHAP values.

The second most significant parameter is LH. For low LH values, there is a noticeable increase in compressive strength, whereas high LH values generally have a negative impact on CS, reducing the prediction. When comparing PS and LH, both features appear to have a similar spread of SHAP values. However, PS is positioned at the top of the feature list, which indicates that it has a slightly higher overall impact on the model’s predictions. This ordering suggests that PS is a more significant parameter compared to LH. The least significant process parameter is PT. The PT feature shows a mixed effect increasing PT values can either climb up or down the output relying on the instance, but generally, low PT values tend to reduce the predicted CS. The results show that the LH clearly demonstrates that low values correspond to higher compressive strength. However, PS and PT exhibit mixed impacts, making it crucial to predict the optimal values for these process input parameters to achieve the maximum CS. The next section will discuss the adjustment of these process variables for improved performance.

### 3.4. Optimization of process parameters for maximum compressive strength

Among the six ML models evaluated, PR demonstrated the best performance in predicting the compressive strength (CS) of multi-layer PLA/AmdPLA functionally graded materials for the FFF 3D printing process. Therefore, the PR model was selected to predict the optimal process parameters for achieving high compressive strength.

**Fig 12:**
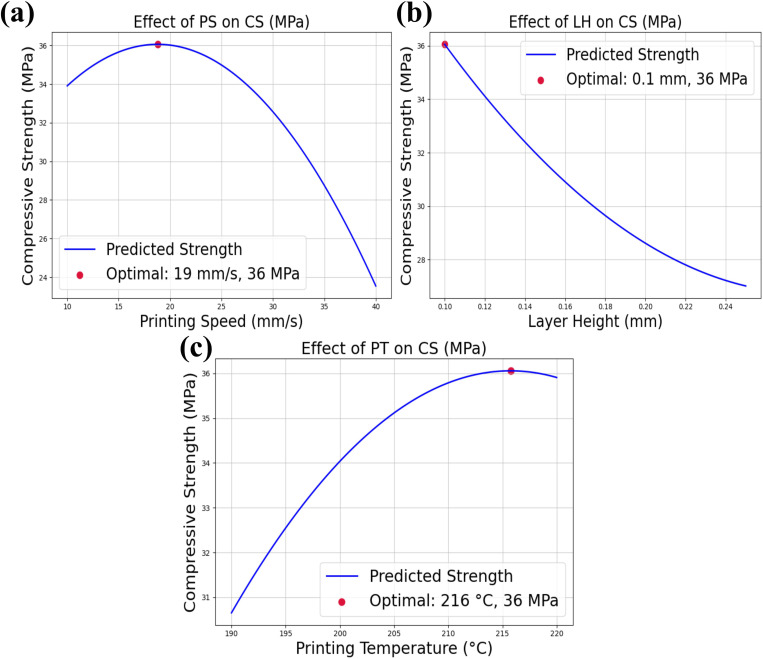
Optimization of result of PR model on process parameters (a) PS, (b) LH, (c) PT.

Fig 12 presents the optimization results using the PR model for three critical process parameters: PS, LH, and PT. In Fig 12a, the optimization results for PS indicate that increasing the PS from 10 mm/s leads to a gradual increase in CS, which reaches its maximum at 19 mm/s. However, beyond this point, the CS begins to decrease. This behaviour can be ascribed to the fact that higher printing speeds tend to reduce the time each layer spends in contact with the nozzle, leading to less bonding between layers and potential defects [40]. Therefore, the optimal value for PS is 19 mm/s, resulting in a maximum CS of 36 MPa, balancing both deposition speed and layer bonding. Fig 12b shows the optimization results for LH. The analysis reveals that as the LH increases, the CS decreases. In contrast, a lower LH results in higher compressive strength. This is because smaller layer heights allow for finer resolution and better layer bonding, which improves the overall durability and strength of the material [41]. Thus, the optimal value of LH is 0.1 mm, which provides the maximum compressive strength of 36 MPa due to improved layer adhesion and minimal voids between layers.

Finally, Fig 12c presents the optimization results for PT. The data suggest that increasing the PT from 190°C leads to a rise in CS, which continues up to 216°C. However, when the temperature exceeds 216°C, the CS starts to decrease. This can be explicated by the fact that elevated temperatures enhance the flowability of the material, leading to better fusion between layers and fewer defects [42]. However, excessively high temperatures can cause thermal degradation of the material, weakening the printed structure. Therefore, the optimal value of PT is 216°C, providing a maximum CS of 36 MPa, ensuring optimal layer bonding without material degradation.

Table 4 presents the comparison between the predicted and experimental CS for the optimal process parameters. The optimal parameters for FFF 3D printing are PS = 19 mm/s, LH = 0.1 mm, and PT = 216°C, with the predicted maximum CS being 36 MPa. To validate the model, specimens were printed using these optimal input parameters, and the CS of the prepared test specimens were measured experimentally, yielding a value of 34.8 MPa. The prediction error for CS compared to the experimental value is 3.44%, which lies within the permissible limits. This demonstrates that the prediction of the PR model provides a reliable projection of the CS for the PLA/AmdPLA functionally graded material.

**Table 4:** Validation of predicted model through confirmation experiments and error analysis.

Sl. No.	Optimal Process Parameters	Predicted CS (MPa)	Experimental CS (MPa)	Error (%)
1	PS-19 mm/s LH-0.1 mm PT-216°C	36	34.8	3.44

### 3.5. Comparison of Taguchi optimization and ML model optimization

**Fig 13:**
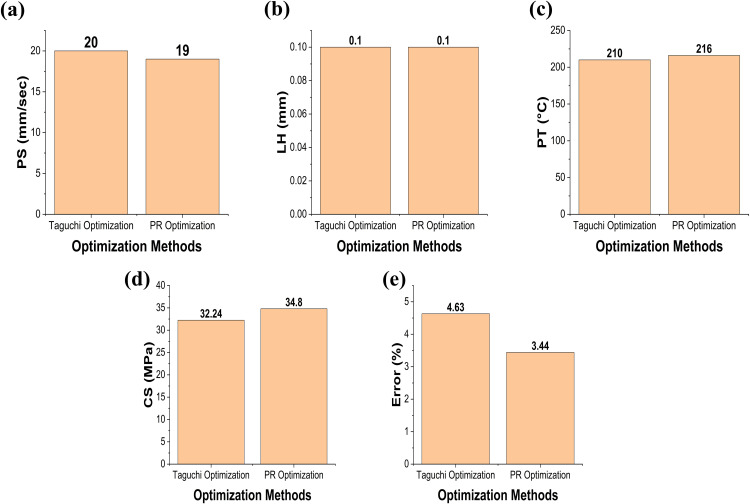
Comparison of Taguchi vs PR (a) PS, (b) LH, (c) PT, (d) CS, (e) error (%).

Fig 13 presents a comparative analysis between the optimal process parameters and the corresponding compressive strength values predicted by the Taguchi optimization method [33] and the PR-based ML optimization method. Fig 13a compares the optimal PS values obtained by both methods. It is observed that the PR model predicts a lower PS than the Taguchi method. A lower PS allows the extruded material to spend more time under the nozzle, improving the interlayer bonding and reducing the chance of defects thereby contributing to better compressive strength [43].

Fig 13b compares the optimal LH values. Both the Taguchi and PR methods predict the same optimal LH value of 0.1 mm. This suggests a consensus between the traditional and ML-based approaches regarding the most favourable layer thickness for achieving high compressive strength. Fig 13c illustrates the comparison of PT. The PR model predicts a higher PT compared to the Taguchi method. A higher PT enhances the flowability of the filament material during extrusion, resulting in improved fusion between layers and fewer internal defects. This improved interlayer adhesion contributes to higher mechanical performance, especially compressive strength [41].

Fig 13d shows the experimental compressive strength values achieved using the optimal parameters from both methods. The specimen fabricated using the PR-optimized parameters demonstrated higher compressive strength than the one produced using the Taguchi-optimized parameters. This further confirms the advantage of using ML for identifying optimal settings that improve mechanical performance. Finally, Fig 13e compares the error percentages between predicted and experimental compressive strengths for both methods. The PR model exhibits a lower error percentage compared to the Taguchi method, indicating that the PR-based ML approach offers a more accurate prediction of the compressive strength for multi-layer PLA/AmdPLA functionally graded materials fabricated using the FFF 3D printing process. Overall, the results demonstrate that the ML-based PR optimization not only identifies more effective process parameters but also results in better mechanical properties and higher prediction accuracy compared to the traditional Taguchi method.

**Fig 14:**
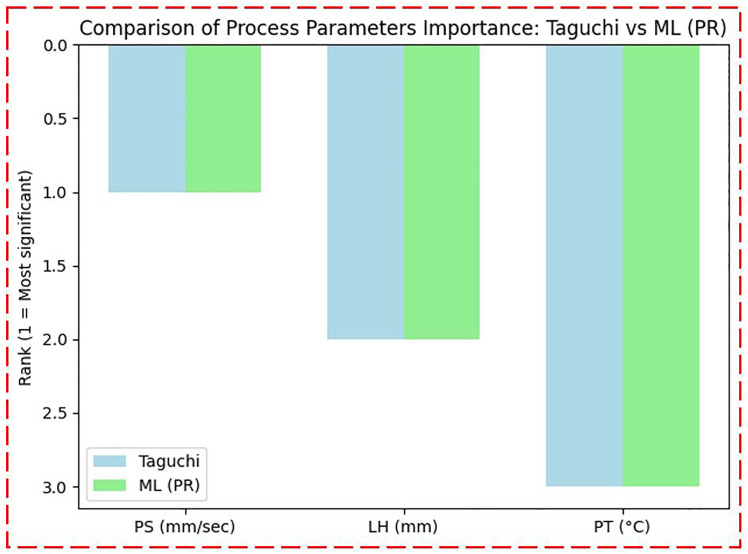
Comparison of process parameters importance: Taguchi vs ML (PR).

Fig 14 shows the comparison of the significance (importance) of process parameters as predicted by both the Taguchi method and the ML method using PR. The comparison shows a strong agreement between the two methods in identifying which process parameters have the most impact on compressive strength. This consistency between a statistical method (Taguchi) and a data-driven method (ML) increases the confidence in the results. Notably, both approaches highlight PS as the key factor affecting the compressive strength of the 3D-printed specimen. This highlights that PS plays a key role in defining the mechanical performance of the multi-layer PLA/AmdPLA functionally graded material produced by the FFF 3D printing process.

## 4. Conclusion

This research work aims to optimize the CS of PLA/AmdPLA multi-layer functionally graded materials in FFF 3D printing by utilizing ML models. The research compares various ML models to predict CS based on key process parameters, including PS, LH, and PT. PR emerged as the most effective model for optimizing these parameters. Finally, ML (PR) was compared with the Taguchi method, and the major findings are as follows:

1. Among all ML models, PR showed the best performance for CS prediction with the highest R^2^ = 0.88, and lowest error metrics (MAE = 1.38, RMSE = 1.9, MSE = 3.6). KNN ranked second (R^2^ = 0.81), while DTs had the weakest performance (R^2^ = 0.55) due to overfitting. The results confirm that PR effectively captures the non-linear relation between process parameters and CS in FFF 3D printing of PLA/AmdPLA composites.
2. The PR, LR, SVR, and KNN ML models demonstrate the good generalization with higher test Cp values than train Cp, indicating no overfitting. In contrast, DTs and RF show lower test Cp, confirming overfitting, with DTs exhibiting the most severe case. Hence, PR remains the most reliable model for CS prediction.
3. SHAP analysis confirms PS as the most influential parameter on CS, showing both positive and negative effects based on its value. LH consistently shows that lower values improve CS, making it the second most critical factor. PT has the least impact with mixed influence.
4. PR model accurately predicted optimal parameters: PS = 19 mm/s, LH = 0.1 mm, and PT = 216°C, resulting in a predicted CS of 36 MPa. Experimental validation yielded 34.8 MPa, with a low error of 3.44%, confirming the model’s reliability.
5. The PR-based ML method outperformed the Taguchi method by predicting 36 MPa compressive strength, compared to 33.74 MPa from Taguchi, showing a 7.5% improvement. The error for the PR model was lower at 3.44%, versus 4.73% for Taguchi. Both methods identified PS as the most influential parameter. Overall, PR-based optimization offers better accuracy and enhanced mechanical performance.

In future investigations, optimizing specimen size with consideration to thermal dissipation effects may further enhance the mechanical properties of the material. Additionally, exploring the influence of controlled or slower cooling during the fused filament fabrication (FFF) process could facilitate the formation of stronger interlayer bonds and a more uniform microstructure. Building on these findings, future research can also focus on integrating deep learning models to improve prediction accuracy and analyse larger datasets encompassing a wider range of process parameters. Moreover, the incorporation of real-time monitoring and adaptive process control during 3D printing holds significant potential for improving fabrication consistency and overall material quality.

### Acronyms

**Table:** 

Artificial Neural Network	ANN
Coefficient of Determination	R^2^
Compressive Strength	CS
Decision Tree	DTs
Extreme Gradient Boosting Regression	EGBR
Genetic Algorithm	GA
Gradient Boosting Regression	GBS
Kernelized Support Tensor Algorithm for Regression	KSTAR
K-Nearest Neighbors	KNN
Linear Regression	LR
Mean Absolute Error	MAE
Mean Square Error	MSE
Multilayer Perceptron	MLP
Pearson Correlation Coefficient	Cp
Polynomial Regression	PR
Random Forest	RF
Root Mean Square Error	RMSE
Support Vector Regression	SVR
Voting Regression	VR
